# Elasticity in Porous 3D-Printed Polylactic Acid Scaffolds for Biomedical Applications: A Predictive Approach

**DOI:** 10.3390/polym17070836

**Published:** 2025-03-21

**Authors:** Matteo Sestini, Dario Puppi, Simona Braccini, Teresa Macchi, Beata Matungano, Sven Macolic, Tommaso Guazzini, Gianluca Parrini, Mario Milazzo, Serena Danti

**Affiliations:** 1 Department of Civil and Industrial Engineering, University of Pisa, Largo L. Lazzarino 2, 56122 Pisa, Italy; matteo.sestini@phd.unipi.it (M.S.); beata.matungano@ing.unipi.it (B.M.); sven.macolic@phd.unipi.it (S.M.); 2 Department of Chemistry and Industrial Chemistry, University of Pisa, Via Moruzzi 13, 56124 Pisa, Italy; dario.puppi@unipi.it (D.P.); simona.braccini@phd.unipi.it (S.B.); 3 Consorzio Interuniversitario Nazionale per la Scienza e Tecnologia dei Materiali (INSTM), via G. Giusti 9, 50121 Florence, Italy; teresa.macchi@phd.unipi.it; 4 Department of Translational Researches and New Technologies in Medicine and Surgery, University of Pisa, Via Risorgimento 36, 56126 Pisa, Italy; 5 L.M.P.E. S.r.l. S.B., Parco Scientifico di Capannori, Via Nuova, 44/A, 55018 Capannori, Italy; tommaso.guazzini@lmpe.eu; 6 Fabrica Machinale, Via Giuntini 13, Cascina, 56021 Pisa, Italy; gparrini@smrobotica.it; 7 The Biorobotics Institute, Scuola Superiore Sant’Anna, Viale Rinaldo Piaggio 34, 56025 Pontedera, Italy

**Keywords:** additive manufacturing, sensitivity analysis, computational materials science, finite element modeling, biomaterials

## Abstract

Additive manufacturing (AM) is rapidly advancing, particularly in biomedical applications, necessitating a deeper understanding of the mechanical behavior of 3D-printed materials. Structures created using fused deposition modeling (FDM) exhibit anisotropic properties due to fabrication inhomogeneity and material architecture. Finite element modeling (FEM) is commonly used to predict mechanical behavior, though studies on porous structures have not deeply investigated the influence of geometrical features on global mechanical behavior. This study aimed to correlate the mechanical properties of porous polylactic acid scaffolds with different patterns and infill densities, fabricated via AM through the synergies of experimental and computational approaches. Tensile testing and FEM simulations were conducted, revealing differences in elastic modulus and tensile strength based on infill orientation. A sensitivity analysis on the main geometrical features assessed variations in filament dimensions and layer spacing. FEM simulations showed strong agreement with experimental data, validating their predictive capability, with deviations due to minor structural defects and irregularities in the extruded filaments. This study established for the first time the influence of geometrical details on the elastic properties of porous scaffolds, opening up to new tailored design for, but not limited to, biomedical applications.

## 1. Introduction

The frequent shortage of bone grafts suitable for repairing defects due to injuries and illnesses has highlighted a critical gap in current medical treatments [1]. While autografts still represent the gold standard due to their osteogenic properties, their use is constrained by the limited availability of donor tissue and patient’s morbidity [2]. Tissue engineering approaches have emerged as a valuable alternative to traditional grafting solutions by offering the possibility to engineer tissues under a personalized medicine approach through customized shapes and structures specifically designed as required in each unique case [3,4].

Additive manufacturing (AM) is considered an effective technological approach, as it allows structurally and dimensionally stable scaffolds to be obtained with complex shapes for addressing the variability of patient-specific anatomical sites [5]. Among AM techniques, fused deposition modeling (FDM) has recently been extensively employed in the biomedical field. FDM utilizes a three-dimensional (3D) printer provided with a heated nozzle to melt thermoplastic materials in a semi-liquid state and thus deposit the formed filament on a layer-by-layer built platform, in which the materials are solidified. This outer device shape is generated based on a specific defect or tissue form, which can be designed or acquired from a patient via computed tomography [6,7].

In the case of bone tissue engineering, AM technologies are able to fabricate porous scaffolds to support bone cell growth, which would ultimately facilitate the biological and mechanical integration of the substitute in the body site [8]. In fact, suitable architectural and mechanical features would entail optimal device biocompatibility by avoiding stress shielding phenomena at the device–tissue interface [9].

However, a relevant challenge to be addressed in 3D printing is the discrepancy in mechanical properties observed between the 3D-printed object and the same bulk material [10]. When using FDM, besides the designed scaffold porosity, the layer-by-layer construction additionally concurs to this issue, as it can introduce a structural anisotropy and irregularities affecting the 3D construct’s mechanical integrity [11]. Moreover, even with equivalent porosity, the inner geometry of the 3D-printed pores, e.g., pore size, distribution and shape, significantly influences the mechanical behavior of the scaffold [12,13].

Therefore, the mechanical properties of 3D-printed scaffolds obtained through FDM have been extensively studied. In particular, key factors directly affecting the geometry and the overall architecture of 3D-printed parts include build orientation [14], build pattern [11,13], layer thickness, raster width and porosity [15]. On the other hand, parameters, such as deposition speed [16,17], extruder temperature [18] and the heated block length [17], which do not directly affect the geometry, also play a crucial role in the resulting mechanical properties. Among the many polymers used in 3D printing, polylactide or polylactic acid (PLA) is a thermoplastic polyester widely used in biomedical applications due to its excellent processability, long-term hydrolytic biodegradation and good biocompatibility, along with appreciable rigidity [19,20,21,22].

Song et al. investigated the anisotropic mechanical behavior of low porosity 3D-printed PLA specimens comparatively to the same specimens obtained through injection molding [14]. By minimizing the porosity of 3D-printed objects, Schiavone et al. further evaluated the effect of printing order, infill patterns and infill direction on tensile properties for two different PLAs displaying different crystallization kinetics [23]. In particular, the 3D850 PLA with relatively slow crystallization rate showed mechanical properties which were more independent of the thermal history that occurred during the printing process due to a better cohesion between layers [23].

In 3D-printed polymer scaffolds and devices that are designed for load-bearing applications, mechanical properties play a major role. Puppi et al. preliminary studied the tensile mechanical properties of 3D-printed specimens made of semicrystalline PLA, with lay-down patterns of 0°/90° and −45°/45° and infill densities of 60% and 100%, finding out that such structures can be suitable to design scaffolds for bone regeneration with tunable mechanical properties [24].

In this context, computational studies are playing a pivotal role in studying and predicting the behavior of these porous structures under various mechanical conditions. To understand the influence of microstructure on the mechanical properties of an object, a multiscale approach can be used: finite element models (FEMs) of representative volume elements were analyzed to extract the macroscale properties by considering the object of interest as a homogeneous orthotropic material [25]. In a similar way, the mechanical properties of PLA microstructures obtained via FDM were investigated by considering variation in the printed filament orientations and flow rate percentages. Moreover, the laminate theory implemented with an elastoplastic constitutive law was applied, in which the micromechanical analysis is triggered only under inelastic strain [26]. Kantaros et al. employed FEM to analyze the compressive mechanical response of porous scaffolds and optimize their design with different structural units [8].

FEM was also employed to optimize medical devices with structural function. For instance, FDM was combined with FEM to optimize the design parameters of a lumbar fusion cage [27]. However, a 3D-printed porous structure was modelled as a bulk and the elastic modulus was determined experimentally for different infill densities. Hence, this approach did not take into account the stress concentrations induced by the microstructure, in particular in the proximity of junction between the printed filaments.

To date, a methodology for predicting and optimizing the mechanical properties of 3D-printed porous scaffolds based on the diversity of specific geometrical features is still missing. Therefore, FEM can serve as a valuable tool to achieve this goal in combination with analytical and experimental methodologies [28].

The present study focused on the study of the tensile elastic mechanical behavior of 3D-printed PLA scaffolds by exploiting the synergy between experimental and computational approaches. Two distinct infill patterns with layers of filaments oriented either at 0°/90° or at −45°/45° with respect to the testing direction were investigated for infill densities of 40%, 60%, 80% and 100%. Afterwards, a comprehensive sensitivity analysis was conducted to explore the effects of process parameters, such as raster width, distance between overlapping filaments and fillet radius (R_f_) on the elastic properties of 3D-printed structures. Eventually, the geometrical variation of the diameter of a single filament was included in the analysis to simulate the non-perfect deposition of the material. This study aims to identify the critical linkage between the geometry of FDM polymeric micropatterned structures and their macroscale elastic mechanical properties to achieve reliable simulation outcomes. Developing reliable in silico models to predict 2D printed scaffold mechanical properties would enable a significant step forward in fabricating complex 3D-printed porous structures, thereby advancing the field of bone tissue engineering.

## 2. Materials and Methods

### 2.1. Experimental Investigation

#### 2.1.1. Materials

PLA (Ingeo Biopolymer 3D850, NatureWorks BV, Naarden, The Netherlands) in the form of transparent pellets, with an average diameter of 2.85 mm, density of 1.24 g/cm^3^, glass transition temperature range of 55–60 °C, and peak melting temperature range of 165–180 °C, was bought from NatureWorks LLC (Minnetonka, MN, USA). The thermal behavior of the 3D850 was comprehensively characterized by Schiavone et al. [23]. Mesenchymal stem cells (MSCs) from human bone marrow were purchased from Promocell (Heidelberg, Germany). StemMACS™ MSC Expansion Medium 130-091-680 was bought from Miltenyi Biotec, Bergisch Gladbach (North Rhine-Westphalia, Germany). Penicillin/streptomycin (PEN-STREP), L-glutamine, Dulbecco’s phosphate buffered saline (DPBS), and resazurin were bought from Merck (Darmstadt, Germany). Fetal bovine serum was purchased from GIBCO (Waltham, MA, USA), while Diflucan and trypsin EDTA were acquired from EuroClone S.p.A. (Milan, Italy). Gelatin, type B from bovine skin was obtained from Sigma-Aldrich (Milan, Italy). Calcein AM was acquired from Life Technologies by Thermo Fisher Scientific (Waltham, MA, USA).

#### 2.1.2. Manufacturing Processes

The Precision 450 filament maker (3Devo B.V., Utrecht, The Netherlands) was employed to fabricate a PLA filament with a diameter equal to 1.75 mm ± 0.05 mm to be used for 3D printing. The temperature of four zones of the extrusion screw section were 170 °C, 190 °C, 190 °C and 170 °C, while the screw rotation speed was set at 5 rpm. A multifunctional AM machine prototype (Fabrica Machinale S.r.l, Pisa, Italy) was used to process the PLA filament through melt extrusion [29]. The .STL file of a 1 mm thick standard specimen, according to ISO-527A-5A [30], was uploaded on the open source Slic3r software (V1.2.9) to generate a G-code file containing the process parameters directly related to the infill pattern and density of the samples. The main printing parameters reported as in [24] are listed in Table 1. Samples were named using the nomenclature *x_y*, in which *x* denotes the infill pattern, either 0°/90° or −45°/45°, while *y* indicates the infill density, with 40, 60, 80 and 100, representing 40%, 60%, 80% and 100% density, respectively.

#### 2.1.3. Optical and Mechanical Characterization

The geometry of the 3D-printed specimens was optically characterized using the Keyence VHX-7000 digital microscope driven by the VHX-H5M software 2.3.16 (Keyence, Milan, Italy).

In particular, the filament length along the *x*- and *y*-axes, the filament length along the *z*-axis and the distance between the filaments were measured separately for geometry 90_y and 45_y with ImageJ software (Version 1.54p): *z*-length (*L_z_*) (*n* = 10) and *x*-/*y*-length of filaments (*L_xy_*) (*n* = 50).

#### 2.1.4. Mechanical Characterization

The mechanical characterization of the 3D-printed specimens was performed by using an INSTRON 5564 (Instron, Norwood, MA, USA) universal testing machine equipped with a 2 kN load cell. ASTM standards D 1708-93 [31] and D 882 [32] were followed; therefore, 1 mm thick standard specimens were fabricated according to the ISO-527A-5A. Five replicates for each printed sample were measured at room temperature (*n* = 5). Stress corresponding to the applied force was computed based on the nominal thickness and width of the specimens.

#### 2.1.5. Physico-Chemical Characterization

Physico-chemical characterization was carried out on the material in the form of pristine pellet, filament and scaffolds. ^1^H-NMR spectroscopy analysis was performed with the JEOL YH 400 MHz spectrometer (JEOL Ltd., Arkishima, Tokyo, Japan). The spectra were recorded on a 2% *w*/*v* polymer solution in CDCl_3_ after 16 scans at 25 °C. ^13^C-NMR spectroscopy analysis was performed with the JEOL CZR 500 MHz spectrometer (JEOL Ltd., Arkishima, Tokyo, Japan). The spectra were recorded on a 15% *w*/*v* polymer solution in CDCl_3_ after 512 scans at 25 °C. Data were processed using the MestReNova software (version 12.0.2).

ATR-FTIR spectroscopy analysis was carried out using a Spectrum 100 FTIR Spectrometer (Perkin Elmer, Waltham, MA, USA) in the wavenumber range of 4000–600 cm^−1^, at a resolution of 4 cm^−1^ and performing 32 scans.

Thermogravimetric analysis (TGA) was performed on samples with a mass of about 5 mg using the Q500 instrument (TA Instruments, Milan, Italy) in the temperature range of 30–700 °C, with a heating rate of 10 °C∙min^−1^, under a nitrogen flow of 60 mL∙min^−1^. The temperature corresponding to the peak maximum on TGA first derivative thermograms (*T_max_*) and the onset temperature of degradation (*T_onset_*) were both recorded on three replicates for each sample (*n* = 3).

Differential scanning calorimetry (DSC) was performed using the Perkin Elmer Instrument (Pyris 1 DSC 6000, Waltham, MA, USA) under a 20 mL/min nitrogen flow. About 10 mg of each sample was placed in a hermetically sealed aluminium pan. It was initially maintained at −20 °C for 1 min, and then it was heated from −20 °C to 220 °C at a rate of 10 °C/min. After reaching 220 °C, it was maintained isothermally for 2 min to erase previous thermal history, followed by cooling from 220 °C to −20 °C at 10 °C/min. The sample was then maintained at −20 °C for 2 min before undergoing a second heating cycle from −20 °C to 220 °C at the same rate. Data were analyzed by means of the Universal Analysis 2000 software (version 4.5A build 4.5.0.5, TA Instruments, New Castle, DE, USA). Melting temperature (*T_m_*) and melting enthalpy (*ΔH_m_*) were determined from the first heating cycle. The fraction of crystalline PLA (*X_c_*), was calculated from the following equation (Equation (1)):

(1) $$X_{c}=\frac{{\Delta H}_{m}}{{\Delta H}_{PLA}^{0}}\times 100$$

where *ΔH^0^_PLA_* is the melting enthalpy of a 100% crystalline PLA, that was considered 93 J/g [23]. The glass transition temperature (*T_g_*) was determined from the second heating cycle to ensure reproducibility and eliminate thermal history effects.

Sample melt flow rate (MFR) was measured with the AMSE Melt Flow Indexer (AMSE S.r.l., Turin, Italy) by applying a 2.16 kg load to a sample with a mass of ~6 g at 190 °C in agreement with the ASTM D 1238-04 standard [33]. The melt flow index mass was determined by measuring the mass of the sample that flowed through a die of 9.61 mm after 10 min.

### 2.2. Computational Modeling

#### 2.2.1. Three-Dimensional Models

The geometrical models of the 3D-printed specimens were designed using the Solidworks 2021 software (Dassault Systèmes, Concord, MA, USA). The flattened shape of the 3D-printed filaments was modeled with a linear slot-shaped cross-section. The joints between filaments of overlapping layers were properly filleted.

**Figure 1:**
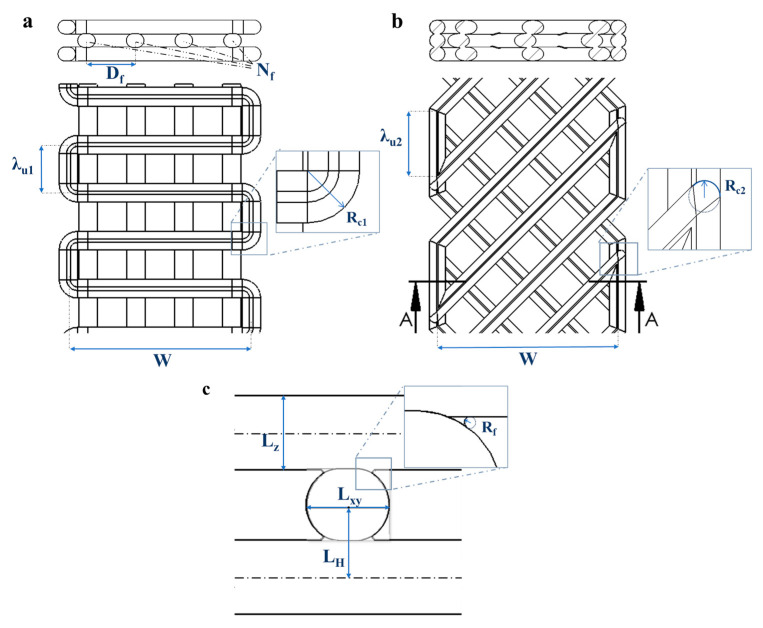
Drawings of the 3D CAD models used to define the 90_y and the 45_y infill patterns. (**a**) Section-view (top) and top-view (bottom) of the 90_y infill pattern with defining geometrical parameters. (**b**) Section-view (top) and top-view (bottom) of the 45_y infill pattern with defining geometrical parameters. (**c**) Welding between overlapping printed filaments with linear slot representing their section.

The geometrical parameters acquired from the 3D-printed structure were used to define the infill pattern (both 0°/90° and −45°/45°) and the dimension of the filament in the computed assisted design (CAD) model, as depicted in Figure 1. In particular, the optical analysis was used to determine the raster width *L_xy_* and the vertical dimension of filament *L_z_* while distances between filaments (*W*, *D_f_*, *λ_u1_* and *λ_u2_*) were determined from the G-code that defines the infill pattern of the different structures.

**Figure 2:**
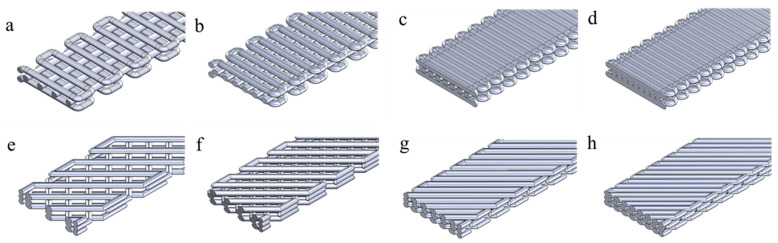
CAD models of infill patterns at 90° (90_y) and 45° (45_y) with the different infill densities: (**a**) 90_40, (**b**) 90_60, (**c**) 90_80, (**d**) 90_100, (**e**) 45_40, (**f**) 45_60, (**g**) 45_80 and (**h**) 45_100.

The CAD models as defined in Figure 2. were designed with different infill densities by varying the geometrical parameters. The geometrical parameters of the CAD models of 90_y and 45_y are listed in Table 2 and reported in Figure 2. In the case of 90_y samples (Figure 2a–d), the structure was composed of three layers: the two external layers consisted of undulated patterns with filaments in transversal direction distanced *λ_u1_*, while the inner layer consisted of a number of longitudinal filaments (*N_f_*), depending on the infill density of the structure.

The CAD models of 45_y presented three layers of filaments tilted with an alternated angle of −45°/45° with respect to the longitudinal direction of the specimen (Figure 2e–h). Therefore, after fixing the width (*W*) of the specimen, it was sufficient to define the distance between filaments along the longitudinal direction (*λ_u2_*) to control the infill density.

#### 2.2.2. FEM Analysis

We employed COMSOL Multiphysics 6.2 (COMSOL Inc., Burlington, MA, USA) to perform a static structural analysis in the elastic range. The PLA was modelled as a linear elastic and isotropic material, setting the Young’s modulus at 2.32 GPa as declared by the supplier.

A preliminary physics-controlled mesh was employed, later refined to achieve the convergence of the results when considering both 90_y and 45_y infill patterns.

To reproduce the actual constraints of the tensile tests, the boundaries at the extremities of the structure were set as prescribed displacement along the tensile test direction and as free along the perpendicular directions. When periodicity and symmetry conditions were applicable, the system was simplified through the selection of a representative volume element (RVE). The tensile force exerted by the structures was obtained by integrating the normal stress field across the section taken at the midpoint of the specimen. Then, to compare the simulated (SIM) versus the experimental (EXP) elastic modulus, the estimated experimental force was divided by the average section of the correspondent 3D-printed specimens.

The predictive accuracy of the elastic modulus was assessed by comparing the prediction error of FEM simulations, denoted as *e_E_*, defined by Equation (2):

(2) $$e_{E}=\frac{E_{SIM}-E_{EXP}}{E_{EXP}}$$

where *E_SIM_* and *E_EXP_* are the elastic moduli obtained via simulation and experimental tests, respectively.

### 2.3. Biocompatibility Tests

#### 2.3.1. Cell Culture

To assess cytocompatibility, the scaffolds (*n* = 3) were sterilized by 97% *v*/*v* ethanol/water overnight, followed by three washings of sterile saline solution. Human MSCs were used to perform the tests. The cells were thawed and suspended in StemMACS™ MSC Expansion Medium, supplemented with 1% PEN-STREP (10,000 penicillin units and 10 mg streptomycin in 1 mL of DPBS), and 1% Diflucan. Cell cultures were carried out in an incubator under standard conditions, namely, 37 °C, 95% relative humidity in a 5% CO_2_/95% air environment. The culture medium was replaced every 2–3 days. Cells were detached with trypsin EDTA, centrifuged for 5 min at 1200 revolutions per minute (rpm), and counted. A filter-sterile 2% (*w*/*v*) gelatin/DPBS solution was used for scaffold pre-coating before seeding. Briefly, the sterile scaffolds were rinsed with the gelatin solution at 100 μL/sample for 5 min, then excess gelatin solution was removed. Afterwards, 250,000 MSCs were seeded for each scaffold. After seeding, cell/scaffold constructs were placed in the incubator for 1 h to allow initial cell adhesion and finally covered with 1 mL of culture medium for each sample. Cell/scaffold constructs were cultured under standard culture conditions for 72 h inside 24-well plates.

#### 2.3.2. Cell Growth

The growth of MSCs cultured on the scaffolds was evaluated in terms of metabolic activity and was monitored throughout the culture time using resazurin dye. Data were acquired according to the manufacturer’s instructions and were expressed as a percentage of reduced dye. The system specifically includes an oxidation-reduction (REDOX) indicator, which exhibits color change in response to a chemical reduction resulting from cell growth. This process causes the REDOX indicator in the medium to shift from its oxidized state (blue, i.e., resazurin) to its reduced state (pink, i.e., resorufin). Results were calculated against negative controls, obtained by adding the dye solution on the scaffold without cells. To perform this metabolic activity test, samples and negative controls (*n* = 3) were incubated for 3 h at 37 °C with the resazurin dye diluted in culture medium according to the manufacturer’s instructions. Viability tests were performed 24 h and 72 h after seeding. At each time point, 100 μL of supernatant from sample or control was loaded in 96-well plates. The absorbance (λ) of supernatants was measured with a spectrophotometer (VICTOR X3 2030 Multilabel Reader; PerkinElmer Waltham, MA, USA) under a double wavelength reading (570 nm and 600 nm). Finally, the reduced percentage of the dye (*D_red_*%) was calculated by correlating the absorbance values and the molar extinction coefficients of the dye at the selected wavelengths. The equation below was applied as follows (Equation 3):

(3) $$D_{red}\%=100\times \frac{\left(\varepsilon_{ox}\right)\lambda_{2}{A\lambda }_{1}-\left(\varepsilon_{ox}\right)\lambda_{1}{A\lambda }_{2}\;of\;sample}{\left(\varepsilon_{ox}\right)\lambda_{2}{A{}^{\circ}\lambda }_{1}-\left(\varepsilon_{ox}\right)\lambda_{1}{A{}^{\circ}\lambda }_{2}\;of\;negative\;control}$$

where: *ε_red_* is the molar extinction coefficient of the reduced form (Red), *ε_ox_* is the molar extinction coefficient of the oxidized form (Blue), *λ*_1_ = 570 nm, *λ*_2_ = 600 nm, *A* is the absorbance of the samples, and *A°* is the absorbance of negative controls.

#### 2.3.3. Cell Adhesion

Adhesion study was performed 24 h after seeding. The samples were treated under live conditions with calcein AM, a non-fluorescent substrate converted to green fluorescent calcein, after acetoxymethyl ester hydrolysis by intracellular esterases. After removing the culture medium, 1 mL of sterile DPBS added with 1 μL of calcein AM was poured in each well and incubated for 30 min at 37 °C in the dark. The results were observed with an inverted microscope equipped for the fluorescence and provided with a digital camera (Nikon Eclipse Ti, Nikon Instruments, Amsterdam, The Netherlands).

### 2.4. Statistical Analysis

Experimental data were reported using the main statistical descriptors. When appropriate, a statistical analysis between groups was performed using the Jamovi software (version 2.3.28) to assess any statistically significant difference between the datasets using a single-way ANOVA with post-hoc Tukey Test, setting a reference probability (*p*)-value equal to 0.05. Matlab R2023a (MathWorks, Natick, MA, USA) was employed to post-process the data.

Data collected from the resazurin assay were evaluated for their parametricity, and a *t* test was performed. For comparisons of the same samples during culture time, a *t* test for paired samples by means was performed; for comparisons between independent samples, based on the variance analysis, a homoskedastic or heteroskedastic *t* test was employed. A *p*-value < 0.05 was set for statistical significance. All data are presented as the means ± standard deviations.

## 3. Results

### 3.1. Optical and Mechanical Characterization of the Scaffolds

**Figure 3:**
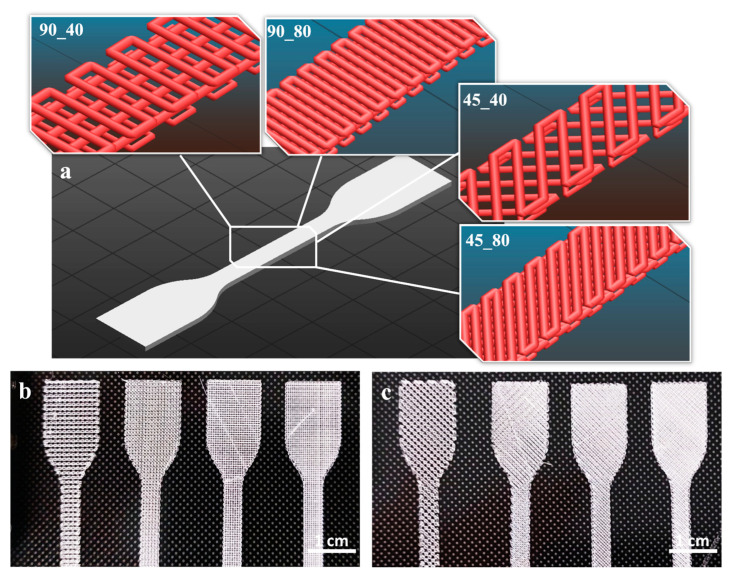
Design and production of 3D-printed specimens. (**a**) Slicing of dog bone specimens with different infill patterns and infill densities. (**b**) Three-dimensional-printed specimens with 0°/90° infill pattern and infill densities of 40%, 60%, 80% and 100%. (**c**) Three-dimensional-printed specimens with −45°/45° infill pattern and infill densities of 40%, 60%, 80% and 100%.

The successful extrusion of PLA filament enabled an effective 3D printing of dog-bone specimens, with infill patterns oriented at 90° and 45° and varying infill densities (Figure 3). To build a representative CAD model reflecting the infill pattern at 0°/90° (90_y) and −45°/45° (45_y), the geometrical parameters of the 3D-printed specimen were characterized by optical microscopy.

The vertical dimension of the printed filament, i.e., *L_z_*, was 295 ± 27 µm, which was coherent to the layer height set in the 3D printing process, i.e., 300 µm. Concerning the 90_y infill pattern, the number of filaments *N_f_* and the distance between longitudinal filaments *D_f_* were defined by the geometry of the sliced specimen. *N_f_* was 4, 6, 7 and 9 for the infill densities of 40%, 60%, 80% and 100%, respectively, while *D_f_* was 1050 µm, 680 µm, 500 µm and 390 µm for the infill densities of 40%, 60%, 80% and 100%, respectively.

The distance between transversal filaments λ_u1_ was determined by dividing the gage length of the dog bone specimen by the number of regions between transversal filaments, and it resulted in 1050 µm, 700 µm, 450 µm and 410 µm for the infill densities of 40%, 60%, 80% and 100%, respectively. *L_xy_* resulted in 376 ± 33 µm.

For the 45_y infill pattern, the geometry is completely characterized by the distance between filaments along the longitudinal direction λ_u2_, which was determined by the geometry of the sliced specimen. It was verified by measuring the distance between parallel filaments and by multiplying it by $\sqrt{2}$. The *λ_u2_* was 1450, 1040, 710 and 560 µm for infill densities of 40%, 60%, 80% and 100%, respectively. *L_xy_* was 334 ± 38 µm.

**Figure 4:**
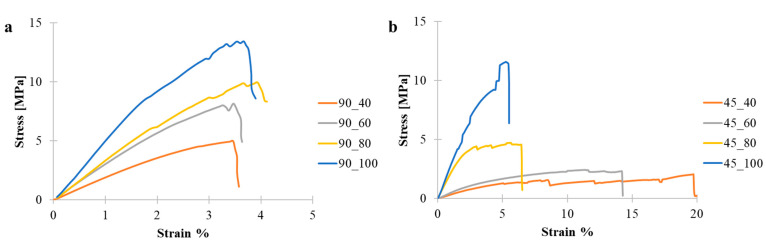
Stress–strain curves obtained from tensile tests on 3D-printed specimens: (**a**) 90_y and (**b**) 45_y specimens.

Therefore, the mechanical properties of 3D-printed specimens with all the combination of infill patterns (0/90° and −45/45°) and infill densities (40%, 60%, 80% and 100%) where characterized. Representative stress–strain curves are shown in Figure 4, and mechanical parameters are provided in Table 3.

Specifically, the 90_y pattern gave rise to stiffer structures, as evidenced by the higher baseline modulus of 198 MPa compared to the 34 MPa of the 45_y specimens, with the elastic moduli increasing from 198 MPa (90_40) to 526 MPa (90_100). In comparison, the 45_y specimens showed a marked increase in the elastic moduli from 34 MPa (45_40) to 294 MPa (45_100). These increases highlighted the significant impact of infill density on enhancing structural rigidity of both 90_y and 45_y specimens.

The tensile strength of the 45_y specimen increased from 1.5 MPa (45_40) to 6.7 MPa (45_100). This trend was similar for the 90_y pattern showing a tensile strength increase from 4.9 MPa (90_40) to 13.5 MPa (90_100). The consistent rise in tensile strength with density underscored the enhanced load-bearing capacity of the specimens at higher densities. The 90_y specimens maintained a comparable elongation at break across all densities, from 3.3% to 3.6%, which suggests that the 90_y orientation contributed to its uniformity, independently of density changes. Conversely, for the 45_y, the elongation at break decreased when increasing the infill density from 13.9% (45_40) to 3.9% (45_100), reflecting a decrease in strain capacity.

### 3.2. Physico-Chemical Characterization of the Material

**Figure 5:**
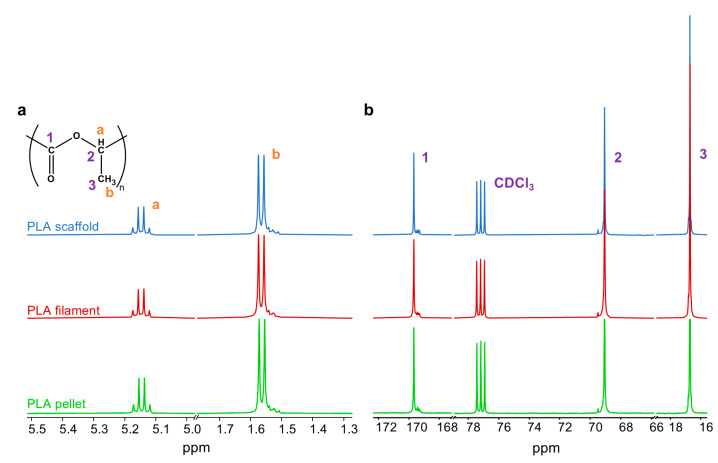
(**a**) ^1^H-NMR and (**b**) ^13^C-NMR spectra of PLA pellet, PLA filament and PLA scaffold with the peaks related to structure shown in (**a**).

The NMR and FTIR analyses were carried out on the PLA pellet, filament and dog-bone specimens in order to assess whether the thermo-mechanical processing steps had an influence on the macromolecular structure. In all ^1^H-NMR spectra (Figure 5a), a signal was visible at around 1.56 ppm indicating the protons of the methyl group of the lactide units (b) and a signal at 5.15 ppm, which indicates the proton of methine group (a). In particular, this last signal consists of a quartet due to different sequences of stereocenters along the polymer chain backbone. This indicates that the investigated PLA is in a D,L stereoisomeric form [34]. ^13^C-NMR spectra (Figure 5b) clearly displayed the characteristic peaks of PLA at 169.67, 69.06 and 16.68 ppm related to the carbonyl, methine and methyl carbons, respectively [35]. ^1^H-NMR and ^13^C-NMR spectra of PLA after each processing step (i.e., extrusion and 3D printing) are superimposable to that of the PLA pellet, suggesting that no appreciable thermal degradation occurred.

**Figure 6:**
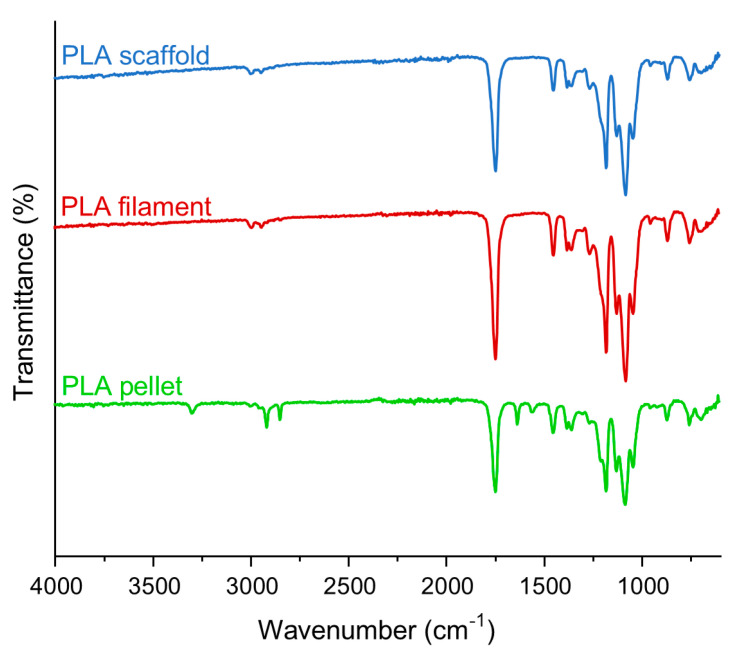
FTIR-ATR spectra of PLA pellet, PLA filament and PLA scaffold.

The FTIR spectrum of PLA before and after processing (Figure 6) is in accordance with what has been previously reported in the literature [36]. In the spectra of the three samples analyzed, characteristic bands are located at 2995 and 2943 cm^−1^, which are related to -CH asymmetric and symmetric stretching vibrations, at 1749 cm^−1^, which is attributed to the stretching of -C=O, 1453 and 1382^−1^ associated with asymmetric and symmetric bending vibrations of -CH, and between 1271 and 1045 cm^−1^, which is ascribed to stretching and bending vibrations of -C-O-C [37]. The comparison of the spectra of the different samples analyzed did not highlight any visible differences related to changes in aliphatic polyester structure due to thermal processing.

**Figure 7:**
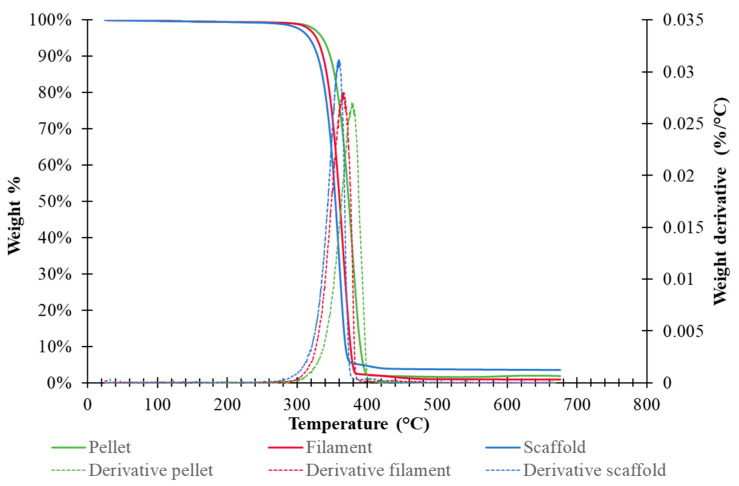
TGA analysis of PLA in the form of a pellet, filament, and 3D-printed filament.

MFR was measured to evaluate the suitability of PLA for FDM. PLA filament MFR obtained through extrusion plastometer analysis was 8.58 g in 10 min. The TGA thermograms of PLA pellet, filament and the 90_60 specimen, analyzed as a representative 3D-printed sample, are reported in Figure 7, while the relevant TGA parameters are resumed in Table 4. The thermogram of the PLA pellet sample showed an onset temperature *T_onset_* of 354.9 ± 3.4 °C and a maximum degradation temperature *T_max_* of 379.2 ± 2.6 °C. The carbonaceous residue was between 1% and 4% of the initial weight (about 5 mg). The PLA filament and PLA scaffold thermogram showed earlier degradation in comparison to the PLA pellet (*p* < 0.001). Partial polymer degradation or changes in its crystallinity degree during screw extrusion can be related to this result. Indeed, previous studies have demonstrated that PLA chain scission can occur under the synergistic effect of temperature and mechanical stress [38].

**Figure 8:**
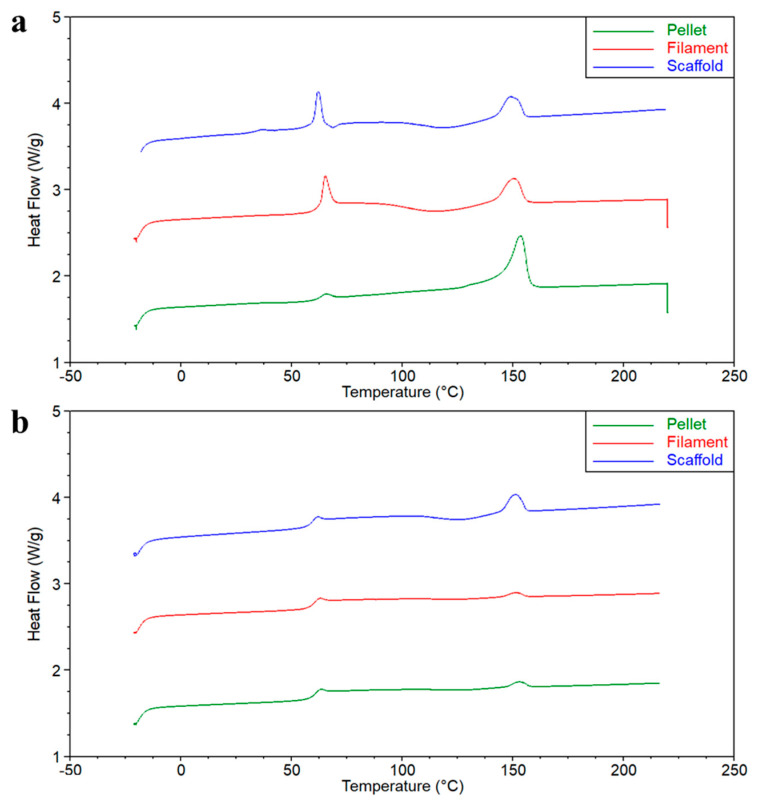
Plot of the DSC curves of PLA pellet, filament and scaffold. (**a**) First heating scan. (**b**) Second heating scan.

The DSC analysis of the PLA pellet, filament and 3D-printed scaffold was performed to investigate their thermal properties. The plots of the first and second heating scan are presented in Figure 8, while the thermal data are summarized in Table 5. In the first heating ramp (Figure 8a), an exothermic peak associated with relaxation was observed between 60 and 65 °C, which falls within the typical range of the glass transition temperature (*T_g_*) for PLA. The relaxation enthalpy (*ΔH_f_*) associated with the peak is higher for the filament and the scaffold, measuring ~10 J/g, whereas for the pellet, it is significantly lower at 2.2 J/g, suggesting a higher fraction of the amorphous phase in a non-equilibrium state for the filament and the scaffold, likely due to differences in thermal history and processing conditions. A distinct melting peak was detected at ~153 °C for the pellet, with a slight decrease in temperature observed for the filament (~150 °C) and scaffold samples (~148 °C). Additionally, the enthalpy of fusion (*ΔH_f_*) was highest for the pellet, while for the filament and scaffold, it was ~20 J/g. Consequently, the estimated crystallinity (*X_c_*) was around 38%, 22% and 20% for the pellet, filament and scaffold, respectively. The observed decrement in crystallinity across the samples may explain the reduction in onset temperature observed in the TGA analysis. The glass transition temperature (*T_g_*) determined from the second heating cycle (Figure 8b) is ~60 °C, with a slight decrease from pellet to scaffold samples.

The peak temperature of enthalpic relaxation and the corresponding relaxation enthalpy are also reported in Table 5.

### 3.3. FEM Analysis

**Figure 9:**
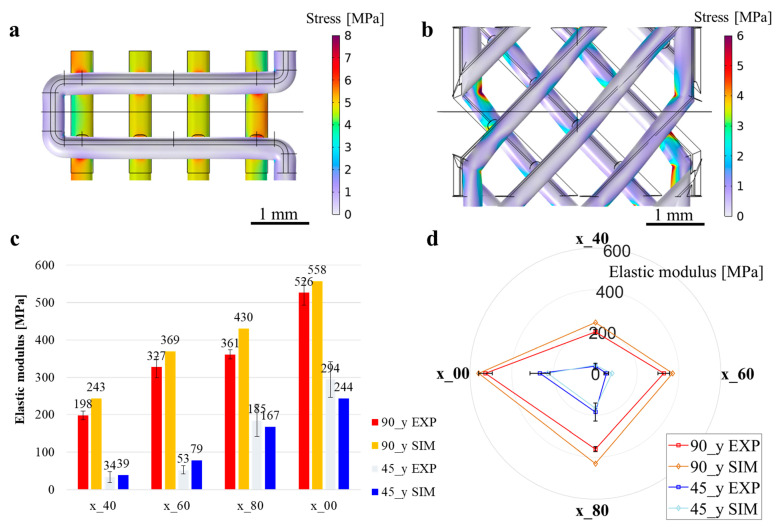
FEM simulations of the 90_y and 45_y structures. Equivalent von Mises stress distribution in tensile mode tested specimens (**a**) 90_40 and (**b**) 45_40, each subjected to a deformation of 0.2%, with overlays showing the undeformed state outlines for reference. Displacements are amplified by a factor of 30. (**c**) Comparison between experimental (EXP) and simulated (SIM) elastic moduli for 90_x and 45_x structures with different infill densities. (**d**) Spider plot for visual comparison between EXP and SIM values of elastic modulus.

FEM analysis was performed to evaluate the influence of the infill patterns and densities on the elastic modulus of 3D-printed specimens and to compare the results between computational (i.e., SIM) and fabricated (i.e., EXP) geometry. Specifically, the simulations focused on infill patterns set at angles of 90° (90_y) and 45° (45_y), with infill densities varying at 40%, 60%, 80% and 100%. The aim was to discern how these parameters affect the mechanical properties, particularly the elastic modulus, of the printed specimens. The von Mises stress distributions of the 90_40 and the 45_40 samples are shown in Figure 9a and Figure 9b, respectively. The computed elastic moduli for each model are reported in Figure 9c. Furthermore, Figure 9c shows the EXP elastic modulus measurements for the same structures. A notable similarity between the SIM and experimentally measured elastic moduli was observed for the 90_y samples, with the simulation values tending to be slightly higher. For instance, in the case of the 90_40 specimen, the experimentally measured elastic modulus was 198 ± 12 MPa, whereas the SIM value was 243 MPa, resulting in a prediction error (*e_E_*) of 23%. In the case of the 90_100 specimen, the computed elastic modulus was 558 MPa, while the corresponding EXP value was 526 ± 33 MPa, yielding a prediction error of 6%.

Instead, for the 45_y samples, the relation between SIM and EXP elastic modulus was variable. The 45_40 yielded an EXP elastic modulus of 34 MPa, which was comparable to the SIM one, i.e., 39 MPa (*e_E_* = 14%). Differently, for the 45_60 specimens, the SIM elastic modulus resulted higher than the EXP one, with an error of 48%. However, at higher densities (45_80 and 45_100) the trend reversed, showing EXP elastic moduli of 185 MPa and 294 MPa, which were higher than the SIM counterparts, namely, 167 and 244 MPa, and a prediction error of 10% and 17%, respectively. This discrepancy highlighted the complex relationship between infill density and mechanical behavior, particularly under different infill orientations. In Figure 9d, the spider plot enables a comprehensive visualization of the mechanical behavior trends across different parameters through the comparison of EXP and SIM elastic moduli.

**Figure 10:**
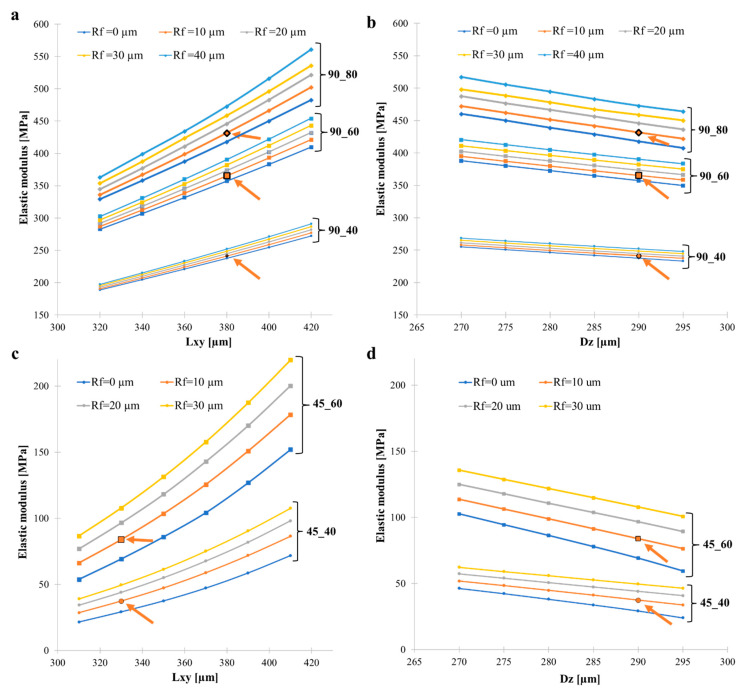
Sensitivity analysis of the 90_y and 45_y 3D-printed structures. (**a**) Elastic modulus variation of 90_y structures as a function of the horizontal length (*L_xy_*) of filaments, parametrized by the fillet radius (*R_f_*), across different infill densities (40%, 60% and 80%). (**b**) Impact of the inter-layer distance (*D_z_*) on the elastic modulus, parametrized by *R_f_* and across different infill densities (40%, 60%, and 80%). Arrows indicate the starting point of the previously SIM architectures. (**c**) Plot describing the relationship between elastic modulus and *L_xy_* of filaments, parametrized by *R_f_*, across infill densities at 40% and 60%. (**d**) Impact of *D_z_* on the elastic modulus, also parametrized by *R_f_* and across infill densities at 40% and 60%.

To deeply investigate the influence of geometric parameters on the elastic mechanical properties of the 3D-printed structures, simulations were iteratively carried out on a selection of the 90_y and 45_y 3D CAD parametrized model, adjusting in the first instance, the dimensions of printed filaments along *L_xy_* and *R_f_*. To this end, a comprehensive parameter sweep was executed for the 90_y, covering all combinations of *L_xy_* ranging from 320 µm to 420 µm in 20 µm increments, alongside R_f_ values set at 0 µm, 10 µm, 20 µm, 30 µm and 40 µm, and combining infill densities at 40%, 60% and 80%. This approach resulted in a total of 90 simulation data points, which are shown in Figure 10a. The elastic modulus of the 3D-printed structures exhibited a positive correlation with the *L_xy_* of filaments, as well as with R_f_. Notably, as the infill density increased, a pronounced enhancement in the increase rate of the elastic modulus with the *L_xy_* occurred. Furthermore, an increase in *R_f_* amplified the impact of *L_xy_* on the elastic modulus, suggesting a synergistic effect between these parameters in reinforcing the structural integrity.

Afterwards, the impact on the 3D-printed structures’ mechanical properties for two geometrical parameters that directly act on inter-filament welding, namely the inter-layer distance (*D_z_*) and *R_f_*, was investigated for infill densities at 40%, 60% and 80%. In particular, the *D_z_* ranged from 270 to 295 µm in 5 µm increments, alongside the *R_f_* values set at 0 µm, 10 µm, 20 µm, 30 µm and 40 µm (Figure 10b). Notably, minor variations in the *D_z_* significantly decreased the elastic modulus, indicating a high sensitivity of the mechanical properties to layer spacing. Similarly to the results shown in Figure 10a, an increase in infill density led to a steeper increase in the elastic modulus across the tested range of the *D_z_* and had a synergistic effect with *R_f_* in influencing the elastic modulus. However, for a specific infill density, the *R_f_* did not exhibit a synergistic effect with the inter-layer distance, as evidenced by the parallel lines in the plots.

A similar analysis was conducted on the 45_y structures. A parameter sweep was executed for the 45_y, covering all combinations of *L_xy_* ranging from 310 µm to 410 µm in 20 µm increments, alongside *R_f_* values set at 0 µm, 10 µm, 20 µm and 40 µm and combining infill densities at 40% and 60% (Figure 10c). As in the 90_y structure, the elastic modulus exhibited a positive correlation with the *L_xy_* of filaments and *R_f_*. As the infill density increased, a pronounced enhancement in the rate of increase in the elastic modulus with *L_xy_* was found. Interestingly, the 45_y demonstrated a more pronounced non-linear relationship between elastic modulus and *L_xy_* compared to the 90_y. Furthermore, *R_f_* amplified the impact of *L_xy_* on the elastic modulus, confirming also for the 45_y structure a synergistic effect between these parameters in reinforcing the structural integrity.

Even in the case of 45_y, the impact of *D_z_* and *R_f_* was investigated to understand the effect of welding between filaments on this type of structure. *D_z_* ranged again from 270 to 295 µm in 5 µm increments, alongside *R_f_* values set at 0 µm, 10 µm, 20 µm and 40 µm for infill densities at 40% and 60% (Figure 10d).

**Figure 11:**
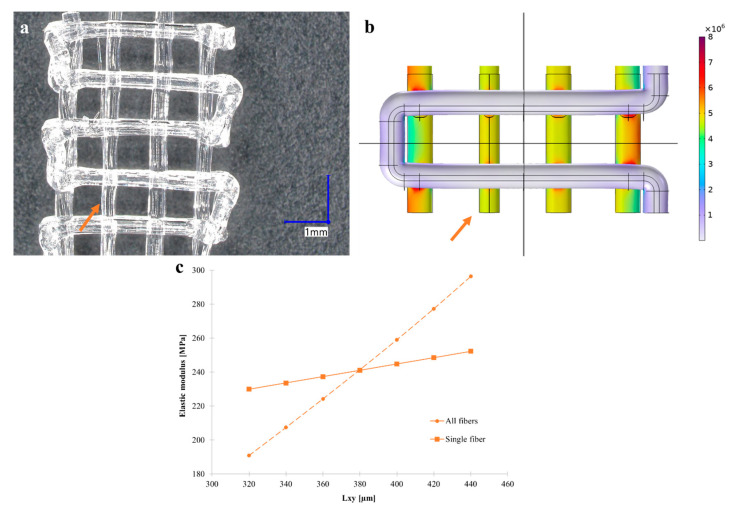
Defect modeling as a reduction in *L_xy_* in a single longitudinal filament. (**a**) Representative stereomicrograph of a tensile tested 90_40 specimen showing the difference in diameter between two parallel longitudinal filaments. The orange arrow refers to a filament with a reduced section compared to others. (**b**) Equivalent von Mises stress distribution in tensile tested 90_40 specimens with reduced filament diameter subjected to a deformation of 0.2%, with overlays showing the undeformed state outlines for reference. Displacements are amplified by a factor of 30. Arrows indicate the altered filaments. (**c**) Decrease in elastic modulus resulting from the selective alteration in *L_xy_* of a longitudinal filament, compared to the reduction in all filaments described with a dashed line.

Notably, the decrement in *D_z_* significantly increased the elastic modulus, indicating a relative higher sensitivity of the mechanical properties to layer spacing. As an example, in the case of 45_40 (*R_f_* = 10 µm) for *D_z_* = 295 µm, *E* = 34 MPa and for *D_z_* = 270 µm, *E* = 52 MPa with an increment of 38%, while for 90_40 the increment was only 3%, highlighting that the structural contouring at the filament junctions played a critical role in 45_y structures. Similarly to the outcomes shown in Figure 11a, an increase in infill density led to a steeper increase in the elastic modulus across the tested range of *D_z_* and had a synergistic effect with *R_f_* in influencing the elastic modulus. However, for a specific infill density, the *R_f_* did not exhibit a synergistic effect with the *D_z_*, as evidenced by the parallel lines in the plots, but for *R_f_* = 0 µm. For higher *D_z_*, the elastic modulus displayed a deviation compared to the other lines.

A similar approach was applied to the parametrized CAD model of the 90_40 structure to accurately represent a specific defect in 3D-printed filaments, specifically targeting variations in the longitudinal filament diameter along *L_xy_*, as it was observed experimentally (Figure 11a).

Hence, the dimension was systematically varied from its original 380 µm size, within a 320–440 µm range with 20 µm steps. In Figure 11b, von Mises stress distribution of 90_40 structure with the selective 340 µm reduction in *L_xy_* in a single longitudinal filament. The outcomes, as depicted in Figure 11c, showcased a marked reduction in the SIM elastic modulus, which is compared to the decrement of elastic modulus by globally reducing *L_xy_*. For instance, the reduction in *L_xy_* in a single longitudinal filament to 340 µm gave a decrease in elastic modulus of about 3%, whereas the reduction in *L_xy_* in the filament diameter of all the filaments resulted in a 14% reduction. This trend underscores the influence of small geometrical alterations on structural integrity and response.

### 3.4. Cytocompatibility Tests

**Figure 12:**
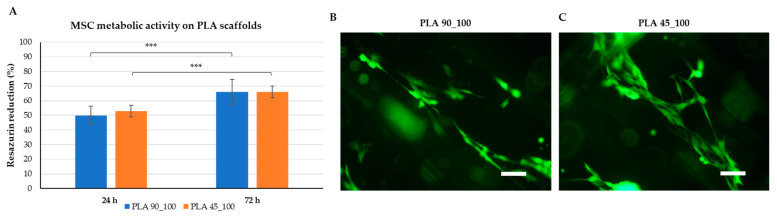
Results of cytocompatibility tests performed by culturing human MSCs on PLA scaffolds for 72 h: (**A**) bar graph showing the metabolic activity of the cells on PLA 90_100 and PLA 45_100 scaffolds at 24 h and 72 h after seeding (data are given as mean ± standard deviation; *** *p* < 0.001); (**B**,**C**) fluorescence micrographs imaging the cells adhered on the surface of (**B**) PLA 90_100, and (**C**) PLA 45_100 scaffolds after 72 h from seeding; scale bar is 100 μm.

The resazurin assay was used to assess human MSC growth on the selected scaffolds in terms of metabolic activity and was performed 24 h and 72 h after cell seeding. Reduction rates identified by the assay are shown in Figure 12A. A metabolic activity enhancement with statistical significance (averagely, 50% and 53% for PLA_100 and PLA_45, respectively, at 24 h, to averagely 66% for both types at 72 h) was observed between the two time points for each type of scaffold (*** *p* < 0.001). Differently, no significant difference was observed between the two scaffolds at the same time points. Figure 12B,C shows live MSCs, elongated and adhered to the scaffold surfaces at 72 h after seeding.

## 4. Discussion

Additive manufacturing (AM) is one of the fastest-growing areas in the manufacturing sector, offering transformative potential for the development of custom-engineered structures across various industries, particularly in biomedical applications [39]. With rapid advancements in additive technologies, there is a growing need to comprehensively understand and predict the mechanical behavior of materials produced through 3D printing to ensure their efficacy and safety in practical applications.

The mechanical behavior of 3D-printed objects fabricated via fused deposition modeling (FDM) is notably anisotropic due to two primary factors:

(i) The inherent fabrication inhomogeneity resulting from the anisotropy of the extruded filament properties along the longitudinal and transverse directions, with the latter being strongly influenced by the properties of filament–filament interfaces [11].
(ii) The architecture of the deposited material, which ultimately determines the load distribution. This aspect becomes increasingly significant as porosity increases [40].

To investigate and predict the mechanical behavior of 3D-printed structures, finite element modeling (FEM) has been widely employed. Specifically, the effect of filament orientation has been studied in low-porosity structures [25,26]. However, only a few studies have focused on using simulation (SIM) to analyze porous structures. While air gaps are typically undesirable in most load-bearing components, engineered porosity is crucial in scaffolds for biomedical applications, particularly in bone tissue engineering. Successful implants depend on bone integration and material degradation [8,41]. In this context, the geometry of deposited patterns plays an increasingly important role in defining the mechanical properties of medical devices.

This study aimed to establish a methodology for predicting the correlation between AM structures and their mechanical properties through a combined experimental (EXP) and computational approach. PLA pellets were extruded to produce filaments, which were then used to fabricate 3D-printed dog-bone-shaped specimens via FDM. The specimens featured different infill patterns (90° and 45°) and infill densities (40%, 60%, 80% and 100%). Each structure was meticulously captured to create a detailed CAD model, subsequently employed in FEM simulations to predict mechanical properties in the elastic region. These simulated results were compared with experimental data. Additionally, a sensitivity analysis was conducted to assess how variations in filament dimensions, layer spacing and Rf influenced model predictions. This integrated approach not only validated the simulation methodology but also provided insight into the variability of mechanical responses in 3D-printed structures, reinforcing the correlation between EXP and SIM mechanical properties.

As an initial step, chemical and physical characterizations of commercial PLA were performed at various processing stages—pristine pellet, extruded filament and 3D-printed scaffold—using nuclear magnetic resonance (NMR) spectroscopy and thermogravimetric analysis (TGA). NMR confirmed the presence of D,L stereoisomeric form, the material stability and the absence of compositional changes during fabrication. The measured melt flow rate (MFR) of PLA in filament form was within the optimal range for FDM, consistent with previous studies [37]. DSC revealed a marked decrease in crystallinity from the pellets to the processed samples (filament and scaffold). This is in line with TGA results that revealed differences in onset and maximum degradation temperatures for PLA across processing stages. Overall, these observations suggest only a minor degradation [42] and confirm the amorphous character of PLA 3D850 [23], in agreement with existing literature.

The microstructures of the 3D-printed dog-bone-shaped specimens were examined using optical microscopy to assess critical parameters such as filament dimensions and undulation spacing, which directly influence mechanical properties. Tensile mechanical testing provided valuable insights into the interplay between infill pattern, infill density and mechanical performance, including elastic modulus, tensile strength and elongation at break.

Notably, specimens with the lowest elongation at break were the 90_y samples, which exhibited higher tensile strength and elastic modulus than the 45_y samples. The latter demonstrated higher elongation at break. A clear trend emerged: for both 90_y and 45_y infill patterns, elastic modulus increased with higher infill density. However, while the 90_y pattern showed a linear increase in elastic modulus, the 45_y pattern exhibited a more complex, non-linear trend. These differences can be attributed to the more intricate deformation behavior of the 45_y samples, involving strain-induced filament alignment in the tensile test direction.

The findings of this study align with prior research by Puppi et al., which investigated 3D-printed PLA structures using similar printing parameters and geometries, though lower elastic moduli were observed in this study [24]. For instance, the elastic modulus of the 90_100 specimen in this study was 530 ± 21 MPa, whereas the reported value for a comparable sample was 1.3 ± 0.1 GPa. This discrepancy may be attributed to differences in PLA material properties and the reduced cross-section of the deposited filaments, which measured an average *L_xy_* of 380 µm in this study compared to approximately 430 µm in the cited work [24].

Porosity plays a crucial role in determining the mechanical properties of 3D-printed specimens and is closely linked to filament length and raster spacing [15]. In this study, each structure was modeled in 3D CAD to incorporate geometrical parameters measured via optical microscopy or derived from the sliced model. Accurate modeling of real structures was essential to achieving good correspondence between experimental and simulated elastic moduli.

The strength of 3D-printed objects depends significantly on interlayer welding, which involves interdiffusion and entanglements across filament interfaces [43]. This process consists of initial contact, neck growth and final coalescence [44]. To geometrically control filament welding, structures were interpenetrated and filleted with a curvature radius [45]. Filleting enhances mechanical integration by ensuring continuity and promoting stress distribution between layers. It was implemented for two primary reasons:

(i) To model the bonding formed when fused material is deposited onto a pre-existing filament.
(ii) To eliminate structural discontinuities that could cause excessive stress concentrations.

Given the periodic nature of the designed structures, manual 3D CAD modeling was feasible due to the limited size of individual structural units. This periodicity also allowed for geometric parameterization, facilitating structural customization for specific applications. For larger or less periodic structures, methodologies capable of automatically reconstructing printed geometries from G-code, as described by Bacciaglia et al., should be incorporated [46]. However, existing algorithms do not currently allow filleting between overlapping filaments, nor do they provide direct control over geometrical parameters.

FEM simulations of 3D-printed structures were conducted under symmetry constraints, applying 3D geometries for both 45° and 90° infill patterns. The inherent repetitiveness of the 3D-printed structures enabled the use of a representative volume element (RVE) approach, allowing a detailed analysis of a small, yet fully representative, structural segment. This approach, combined with a linear elastic model, minimized computational complexity while maintaining analytical accuracy.

Despite some variations between SIM and EXP elastic modulus values, particularly for the 45_60 sample, the overall agreement was strong. For both the 90_y and 45_y patterns, FEM overestimated elastic modulus at low infill densities but achieved the best convergence at higher densities. These findings validate the effectiveness of FEM simulations in capturing the essential mechanical properties of 3D-printed structures, supporting the potential of AM PLA structures for load-bearing applications in tissue engineering.

However, experimental results exhibited variability, likely due to uncertainties in filament–joint geometry and printing-induced defects. A sensitivity analysis incorporating geometrical fluctuations through FEM helped interpret this variability.

The influence of *L_xy_* was particularly significant for 45_y structures, where a non-linear relationship with elastic modulus was observed. Additionally, Rf played a crucial role in determining elastic moduli, interacting with *L_xy_* to affect mechanical performance. Variations in layer thickness (*D_z_*) also impacted mechanical properties, though no synergistic effect between *D_z_* and *R_f_* was detected.

This study established a framework for correlating 3D-printed structures with their mechanical properties across a wide range of elastic moduli. Such insights enable the design of PLA unit cells tailored for various biomedical applications and in particular personalized bone defect treatments.

In fact, a crucial aspect of bone defect treatment lies in matching the mechanical properties of the obtained scaffolds to those of biological tissues with similar characteristics. According to the experimental data analyzed in the present work, the mechanical properties of the fabricated scaffolds varied depending on the architectures (i.e., 45/90) and porosity. The elastic moduli ranged from ~30 MPa to ~500 MPa, while the tensile strength ranged from ~1.5 MPa to ~13.5 MPa. In contrast, the elongation at break varied from ~14% for the softest architecture to ~3% for the stiffest one.

Trabecular bone could be a key target for this work, as the scaffold mechanical properties align well with those of this specific tissue. The elastic modulus with values between 100 MPa and 500 MPa was reported for trabecular bone samples from specific anatomical sites, including the vertebra, greater trochanter and femoral tibia. Additionally, the tensile strength in the human trabecular bone was reported to vary between 2 MPa for vertebral trabecular bone and 7 MPa for distal femoral bone [47,48].

Mosekilde et al. also characterized the mechanical properties of vertebral trabecular bone along the vertical direction and reported a value of about 67 MPa for the elastic modulus, 2.45 MPa for the tensile strength, and an elongation at break of 7.4% [49]. Based on our data, the scaffold here analyzed with a 45° architecture and a density around 60% reasonably fell within this range, becoming a potential candidate for bone vertebral applications. Preliminary biological tests conducted at 72 h showed that the proposed scaffolds also supported the in vitro growth of human MSCs, which adhered well to the printed filaments, overall suggesting a potential for their application as regenerative and supporting scaffolds in specific bone defects.

Future work will extend this model to account for non-linear mechanical behavior, incorporating viscoelastic properties of thermoplastics such as PLA. Enhancing predictive accuracy in this manner will expand the applicability of AM structures in biomedical engineering, facilitating the development of implants that closely mimic natural bone mechanics. Such improvements are crucial for successful graft integration, promising better outcomes in bone repair and reconstruction.

## 5. Conclusions

In this study, we investigated FDM structures fabricated via 3D printing with varying infill patterns (0/90°, −45/45°) and densities (40%, 60%, 80% and 100%) using experimental (EXP) and simulation (SIM) techniques. The objective was to develop a predictive model for the mechanical properties of inherently porous scaffolds designed for bone tissue engineering. The collected data covered a broad range of elastic moduli and elongations at break. Finite element modeling (FEM) proved to be a valuable tool for predicting the mechanical properties within the elastic region of 3D-printed PLA specimens with different infill patterns and densities. The EXP results and SIM analyses were generally in good agreement; however, some discrepancies were observed, which could be attributed to certain limitations, such as the approximation of the material as isotropic and elastic, the presence of defects and inhomogeneities in the 3D-printed structure, and incomplete knowledge of the mechanical behavior and geometry of filament–filament welds.

Sensitivity analysis revealed that several geometric parameters significantly influenced the elastic properties of 3D-printed PLA structures. *L_xy_* had a critical impact, particularly in 45_y structures, where a non-linear relationship with the elastic modulus was observed. *R_f_* also played a significant role, interacting synergistically with *L_xy_* at filament junctions and affecting the overall structural performance. Additionally, variations in *D_z_* strongly influenced the elastic modulus, as it was directly related to interlayer bonding. Furthermore, the impact of defects, specifically in the form of localized filament diameter restrictions, was quantified.

Overall, this study provides a solid foundation for further research into optimizing 3D-printed structures for tailored mechanical applications in load-bearing scaffolds for tissue engineering. It also highlights opportunities for improvement in both material properties and modeling techniques.

## Figures and Tables

**Table 1:** Parameters used for PLA 3D printing.

Parameter	Value
Nozzle diameter	400 µm
Layer height	300 µm
Infill speed	10 mm/s
Extruder temperature	210 °C
Bed temperature	40 °C
Infill density	40, 60, 80 and 100%
Infill pattern	0°/90° and −45°/45°

**Table 2:** CAD model geometrical parameters of the infill pattern at 90° (90_y) and 45° (45_y) for the different infill densities.

General Parameters	Structure	Draw Parameters	Volume [mm^3^]	Geometry
L_xy_ = 0.38 mm L_z_ = 0.30 mm L_H_ = 0.29 mm R_f_ = 0.01 mm R_c1_ = 0.195 mm W = 3.94 mm	90_40	N_f_ = 4 D_f_ = 1.05 mm λ_u1_ = 1.05 mm	32.88	a
	90_60	N_f_ = 6 D_f_ = 0.68 mm λ_u1_ = 0.7 mm	46.53	b
	90_80	N_f_ = 7 D_f_ = 0.5 mm λ_u1_ = 0.45 mm	66.48	c
	90_100	N_f_ = 9 D_f_ = 0.39 mm λ_u1_ = 0.41 mm	75.26	d
L_xy_ = 0.33 mm L_z_ = 0.30 mm L_H_ = 0.29 mm R_f_ = 0.01 mm R_c2_ = 0.1 mm W = 3.94 mm	45_40	λ_und_ = 1.45 mm	29.93	e
	45_60	λ_und_ = 1.04 mm	39.30	f
	45_80	λ_und_ = 0.71 mm	55.37	g
	45_00	λ_und_ = 0.56 mm	67.69	h

**Table 3:** Mechanical properties of the 3D-printed PLA structures.

Infill Pattern_Density (x_y)	Elastic Modulus [MPa]	Tensile Strength [MPa]	Elongation at Break (%)
**90_40**	198 ± 12 ^a^	4.9 ± 0.1 ^a^	3.3 ± 0.4 ^a^
**90_60**	327 ± 28 ^b^	8.3 ± 0.8 ^b^	3.5 ± 0.2 ^a^
**90_80**	361 ± 12 ^b^	10.4 ± 1.7 ^b^	3.5 ± 0.2 ^a^
**90_100**	526 ± 33 ^c^	13.5 ± 2.2^c^	3.6 ± 0.5 ^a^
**45_40**	34 ± 15 ^a^	1.5 ± 0.4 ^a^	13.9 ± 5.7 ^a^
**45_60**	53 ± 11^a^	2.4 ± 0.4 ^a*^	13.4 ± 2.5 ^a^
**45_80**	185 ± 43 ^b^	4.1 ± 1.1 ^b*^	5.5 ± 1.0 ^b^
**45_100**	294 ± 48 ^c^	6.7 ± 2.0 ^c^	3.9 ± 0.9 ^b^

Data expressed as mean ± standard deviation (*n* = 5). For each mechanical property and structure (45_y or 90_y), data referred with the same symbols (a,b,c,*) are significantly different between the others but not between themselves. If two symbols are present (i.e., a*), one of the two symbols in common denotes overlapping group memberships.

**Table 4:** Data relevant to the TGA for PLA in the form of a pellet, filament and scaffold.

	Pellet	Filament	Scaffold
**T_onset_ [°C]**	354.9 ± 3.4 **	342.4 ± 1.4	340.2 ± 1.8
**T_max_ [°C]**	379.2 ± 2.6 **	365.6 ± 0.5	362.4 ± 3.8

Data are reported as mean ± standard deviation. ** denotes a value which is statistically different between all the other values (*p* < 0.001).

**Table 5:** DSC data. *T_m_*: peak temperature of the melting process, *ΔH_m_*: enthalpy of fusion, *X_c_*: fraction of crystalline PLA calculated by Equation (1), *T_g_*: glass transition temperature from second heating scan, *T_relax_*: peak temperature of relaxation process, *ΔH_relax_*: enthalpy of relaxation process.

	Pellet	Filament	Scaffold
**T_m_ [°C]**	153.3	150.0	148.4
${\Delta H}_{m}$ **[J/g]**	35.1	20.5	18.2
**X_c_ [%]**	38	22	20
**T_g_ [°C]**	61.1	60.8	60.0
**T_relax_ [°C]**	65.4	65.2	62.1
${\Delta H}_{relax}$ **[J/g]**	2.2	9.7	9.5

## Data Availability

The raw data supporting the conclusions of this article will be made available by the authors upon request.
